# Exploring current approaches towards patient prioritisation for clinical pharmacy services in UK mental health inpatient care

**DOI:** 10.1186/s12888-025-06956-4

**Published:** 2025-07-01

**Authors:** Fatima Q. Alshaikhmubarak, Richard N. Keers, Petra Brown, Penny J. Lewis

**Affiliations:** 1 Division of Pharmacy and Optometry, The University of Manchester, Manchester, UK; 2 NIHR Greater Manchester Patient Safety Research Collaboration, Manchester, UK; 3 Optimising Outcomes With Medicines (OptiMed) Research Unit, Pennine Care NHS Foundation Trust, Manchester, UK; 4 Manchester University NHS Foundation Trust, Manchester, UK

**Keywords:** Patient prioritization, Acuity, Mental health, Psychiatry, Inpatient, Clinical pharmacy

## Abstract

**Background:**

Mental health services in the United Kingdom (UK) are experiencing heightened pressures due to an increase in the number of patients accessing secondary mental health services amidst a period of workforce and funding constraints. Patient prioritisation for inpatient clinical pharmacy services is a promising approach to delivering optimal services with limited available resources. Research has identified several patient prioritisation tools used by acute care pharmacists across the UK, yet little is known about the use of pharmaceutical patient prioritisation approaches in mental health settings. Therefore, this study aimed to explore patient prioritisation approaches used by UK inpatient mental health pharmacy teams.

**Method:**

An electronic national questionnaire was distributed among chief/senior pharmacists within all UK mental health organisations to identify the use of patient prioritisation systems (tools or processes). This was followed by semi-structured interviews with representative members from organisations that responded to the questionnaire and reported using prioritisation systems, and document analysis for prioritisation systems if shared. Interviews were transcribed and analysed using thematic analysis as described by Braun & Clarke. Ethical approval was obtained from the University of Manchester Ethics Committee.

**Results:**

A 75.3% (*n* = 55/73) response rate was achieved for the questionnaire between July 2022 and January 2023. Of organisations that completed the questionnaire, 38.2% (*n* = 21/55) used a prioritisation system within their inpatient pharmacy services in England (*n* = 14/21), Scotland (*n* = 6/21), and Northern Ireland (*n* = 1/21). Sixteen staff representatives from 15 organisations reporting the use of prioritisation systems were interviewed, and 11 prioritisation documents were received and analysed. Prioritisation systems varied greatly in their complexity and development approaches, and the majority were simplistic in design, developed solely based on existing expertise, and lacked formal evaluation. However, all prioritisation systems were perceived by staff to be beneficial in improving patient safety, standardising care, and optimising clinical pharmacy services. Prioritisation criteria included some high-risk medicines (e.g. lithium), issues related to mental health legislation, and patient related factors such as swallowing difficulties.

**Conclusion:**

This study identified 21 UK-based mental health organisations using systems to prioritise inpatients for clinical pharmacy services. Patient prioritisation systems were reported to be beneficial for pharmacy teams in managing their workload and delivering services despite their reported limitations. However, interviews with a single representative may not fully capture the perspectives and experiences of others.

**Supplementary Information:**

The online version contains supplementary material available at 10.1186/s12888-025-06956-4.

## Background

Globally, mental health services are subject to resource constraints [1]. In the UK, ongoing efforts are aimed at achieving parity of esteem with acute care services by raising the level of funding for mental health services [2]. However, this remains unattained [3], and the investment is expected to be insufficient to address the increasing demand for services [4]. This along with ongoing staff shortages [5] and the increased number of adults accessing secondary mental health services [6, 7] highlight the need for efficient use of limited resources [8, 9].

Clinical pharmacists are an integral part of mental health services where medications are the main therapeutic intervention and patients may be highly vulnerable to medication harm due to multi-morbidity [10] and conditions like cognitive impairment [11, 12]. Clinical pharmacists significantly contribute towards patient safety through reducing drug related problems [13, 14] which are widely recognised as a substantial cause of patient harm [15] and extra costs [16]. Drug related problems are highly preventable in mental health with a systematic review reporting a medication error rate of 10.6–17.5 per 1000 patient-days and an adverse drug event rate of 10.0–42.0 per 1000 patient- days [17]. The delivery of optimum pharmaceutical care is, however, complicated by the aforementioned staff shortages and service delivery challenges. Additionally, the shift towards community services such as general practice, care homes, and Primary Care Networks (PCNs) may draw pharmacists out of the hospital exacerbating the staff shortages [18].

Optimal deployment of pharmacy services is a practical strategy proposed by a 2018 NHS report exploring how to improve productivity in mental health services [19]. It was proposed to improve patients’ health outcomes and to mitigate resources constraints. Additionally, the Royal Pharmaceutical Society has emphasised the need for treatment optimisation through directing pharmaceutical services to patients with high risk medications [20]. With prioritisation tools now well established in nursing practice [21, 22] with one tool specific for mental health care called the Mental Health Optimal Staffing Tool (MHOST) [23], patient prioritisation has emerged as a promising approach to deploy pharmacy services [24]. However, unlike the MHOST that uses patient acuity and dependency data to inform workforce allocation, pharmaceutical prioritisation tools prioritise individual patients for pharmacy review reflecting the operational priorities within each profession.

In 2018, an international systematic review of pharmaceutical patient prioritisation tools identified 17 tools from around the world, none of which was specifically designed for mental health care [25]. This review highlighted the perceived impact of these tools on the provision of acute care pharmacy services and patient care [25]. More recently, a UK survey of NHS trusts reported that 54% of hospitals used pharmaceutical patient prioritisation approaches within an acute care setting [24].

Within the recent emergence of research on patient prioritisation for pharmacy services, mental health has received notably limited attention. The use of acute care tools in mental health may be impractical considering unique medication related issues in mental health [26] and factors related to UK mental health law necessitating different prioritisation criteria. For example, high doses and combination therapy of antipsychotics are frequently used [26] which increases the risk of medication related problems whereas the use of intravenous medications is infrequent. Furthermore, non-adherence to medication is common in mental health units [26]. Hence, understanding current pharmacy patient prioritisation practices and existing prioritisation criteria in mental health inpatient care is key to guide future research aiming to develop interventions or tools to optimise service delivery. Therefore, this study aimed to explore the configuration, implementation and impact of current approaches for prioritising inpatient clinical pharmacy services in UK mental health trusts and boards.

## Methodology

This study consisted of a national questionnaire followed by semi-structured interviews and document analysis.

### Questionnaire

An electronic questionnaire [Additional file.1] was emailed to chief/senior pharmacists from all UK mental health trusts and health boards that provided inpatient mental health care using the questionnaire tool Qualtrics [27]. A total of 73 mental health organisations in the UK providing inpatient mental health services were identified through internet search [28–32] and the research team networks. Names and contact details of chief/senior pharmacists were obtained through searching the internet, directly contacting some organisations, and the research team’s professional network. An email invitation explaining the purpose of the study along with a link to the electronic questionnaire was sent during July 2022. Up to 2 reminders were sent to non-responders two weeks after the first email. Some reminders included the response rate and median completion time as this has been previously shown to increase the response rate [33]. Additionally, the study was advertised at the 2022 College of Mental Health Pharmacy conference. Respondents who were unsure or unclear as to whether their approach to prioritisation was considered a prioritisation system were contacted for further clarification.

The questionnaire [Additional file.1] was developed and internally piloted within the research team to ensure clarity, appropriateness of language, and logical flow. It aimed to identify organisations where systems (tools or processes) to prioritise inpatients for clinical pharmacy services were used. Based on answers to the main question “Do you use any system (process or tool) to prioritise inpatients for pharmaceutical care or clinical pharmacy services within your organisation? This could be in all or parts of your organisation, such as a specific ward or specialty”, respondents were directed to different questions. Choosing “yes” or “other” directed them to questions about the type of prescribing (i.e. electronic or paper based prescribing) in their organisation, the type of prioritisation system, its introduction and evaluation. They were also asked if they were willing to be contacted for interview by the research team in future. Respondents who responded with “no” were directed to questions about possible future use and barriers to implementing a system. Last, respondents who chose “We have used a system before, but currently do not” were asked for further details. All respondents were asked basic demographic information.

A prioritisation system was defined as any guidance for pharmacy team members in prioritising patients which can be a process or a tool. A process can be a simple rule or a more complex process that includes a series of steps that guide pharmacy team members in approaching hospitalised patients. A tool can be electronic or paper-based and might include a list of high-risk indicators. A broad definition was used to capture the different levels of complexity in pharmaceutical patient prioritisation systems as this is the first study to explore such approaches in UK mental health organisations.

### Interviews & document analysis

Questionnaire respondents who indicated that prioritisation systems were used in their organisations were invited to take part in semi-structured online interviews, conducted via Microsoft Teams, to learn more about their systems including development, implementation, evaluation and validation, alongside their experiences of their use. We aimed to interview representatives from all organisations to gain better understanding of existing prioritisation systems. Key-informant sampling was employed with up to two members of pharmacy staff (either individually or as a group) with direct knowledge of the prioritisation system interviewed from each organisation. This was a member of staff of any seniority or status (e.g. pharmacy technician, pharmacist). Interviews were conducted by FQA and an interview guide, based on previous research [24], [Additional file.2] was used to direct the interviews. It was piloted in the first three interviews which were included in the analysis as no amendments were required. An information sheet was sent to all participants and informed consent was sought prior to interviews. At the end of the interview, participants were asked whether they consented to waive anonymity on behalf of their organisation. Participants were asked to email copies of their prioritisation systems and related documents after scheduling an interview to enable identification of system characteristics as well as to assist in adapting the interview questions.

### Data processing & analysis

Questionnaire results were summarised using descriptive statistics via Microsoft Excel. Open ended questions underwent thematic analysis with the aid of NVivo software. Questionnaire responses were edited to accurately reflect usage of patient prioritisation systems based on additional information obtained through open-ended questionnaire questions, interviews, or direct contact with respondents via email or phone calls.

Interviews were conducted by FQA, a PhD student, and were video recorded and transcribed by a university approved professional transcribing service before being double checked prior to analysis. The first two transcripts were analysed by FQA and PJL to identify any problems with probing or the use of leading questions, consequently improving the interviewer’s skills [34]. Document analysis and interview analysis were conducted simultaneously using a reflexive thematic analysis method described by Braun and Clarke [35]. After about eight interviews an initial coding framework was developed and consistently applied to the remainder of the interviews. Analysis was undertaken by FQA with continuous supervision and discussion with the research team. All transcripts were independently reviewed by PJL and RNK and a detailed analysis and refinement of themes was undertaken until clearly defined themes were achieved.

### Researcher reflexivity

The research team comprised pharmacists with clinical and academic backgrounds which positioned them to engage meaningfully with participants and interpret data within the appropriate professional context. FQA was a PhD student who at the time had no previous experience with qualitative research. However, she completed relevant training and workshops in qualitative interviewing, thematic analysis, and NVivo software to build the necessary skills. Her position as an ‘outsider’—with international pharmacy experience and no prior involvement in UK mental health settings—offered a fresh perspective and reduced the risk of preconceptions influencing data interpretation. To better understand the context, she shadowed a UK mental health hospital pharmacist for one day.

PJL (senior clinical lecturer in pharmacy), RNK (mental health pharmacist and a senior clinical lecturer in pharmacy), and PB (chief pharmacist at a mental health trust and an honorary reader) are all pharmacists with experience in UK hospitals and of qualitative research studies resulting in multiple publications. They supported FQA throughout the conduct and analysis of this research and held regular discussions throughout data collection and analysis to ensure methodological rigour.

## Results

Of the 73 mental health organisations contacted, 55 (75.3%) representative respondents completed the questionnaire. A total of 21 (38.2%) organisations were determined to be using a prioritisation system and 16 staff members from 15 (71.3%) organisations were interviewed between August – December 2022. The interview duration ranged from 26 to 63 min (mean = 38.7 min). Eleven participants shared the patient prioritisation systems used within their organisations for document analysis.

### Questionnaire

A total of 57 questionnaire responses from 55 organisations were received, of which 22 reported using a system, 21 reported not using a system, 11 chose ‘other’, and only one respondent mentioned using a system before that is no longer used. After analysing free text responses and contacting respondents for further clarification, 38.2% (*n*= 21/55) were determined to be using a prioritisation system and none used a system before but no longer uses one. A summary of the responses can be found in Table 1. Organisations reporting that they were not using a prioritisation system (*n* = 21/55) explained, via free text responses, that they prioritised services using clinical judgment with no ‘official’ system, by reactive approaches (i.e. responding to problems as they arise rather than proactively anticipating and planning), or that they prioritised new admissions or specific wards.

**Table 1:** Numbers and percentages of responses and system usage broken down by country

**Country**	No. of organisations approached	No. of organisations responded	Response rate	Responding organisations using prioritisation systems		
				**Number**		**Percentage**
Wales	7	7	100%	0		0%
Scotland	12	11	91.6%	6		54.5%
Northern Ireland	5	4	80%	1		25%
England	49	33	67.3%	14		42.4%
Total	73	55	75.3%	21		38.2%

Respondents from organisations where prioritisation system were not used were asked whether they were considering or planning to implement one and 66.7% (*n* = 14/21) responded with ‘maybe’, 28.6% (*n* = 6/21) responded with ‘no’, and one (*n* = 1/21, 4.8%) responded with ‘yes’. From thematic analysis of free text responses associated with this question, the majority of respondents who chose ‘no’ (*n* = 5/6) did not oppose the idea. Respondents explained that they did not have sufficient workforce, had not considered the idea before, or did not find a readily available system to use. One believed it might be useful for larger teams or teams with no specialist knowledge in mental health such as rotational pharmacists (i.e. pharmacists who rotate between specialities and may often be junior). Only one respondent was sceptical about prioritisation systems due to the challenge in achieving accuracy when prioritising patients.

Several respondents who reported not using a system linked prioritisation systems with electronic prescribing and stated that they would look to develop or use a prioritisation system as they move to electronic prescribing.

Questionnaire respondents highlighted several barriers to the use of a prioritisation system. These included staff shortages, lack of a standardised prioritisation system, limited funding, and competing priorities.

**“ …** Short staff [staff shortages] provides a barrier to implementing something that requires development first. A standardised tool would seem more sensible than developing different tools.” (S. 48).

Organisations which reported using a prioritisation system were asked for more information, a summary of which can be found in Table 2. Respondents were given the opportunity to add free text comments or feedback at the end of the questionnaire and some provided further details of their system, whereas others expressed their interest in the topic, in seeing other prioritisation tools, and in developing or utilising a prioritisation system.

**Table 2:** Details of currently used prioritisation systems

	No. and percentage of organisations (*n* = 21)
Type of prioritisation system:	
Electronic	7 (33.3%)
Paper-based	8 (38.1%)
Both	6 (28.6%)
Type of inpatient prescribing at their organisation:	
Electronic	12 (57.1%)
Paper-based	6 (28.6%)
Both	3 (14.3%)
Duration of use of prioritisation system:	
Under development	2 (9.5%)
< 1 year	4 (19%)
1—5 years	10 (47.6%)
6—10 years	3 (14.3%)
> 10 years	1 (4.8%)
I do not know	1 (4.8%)
Evaluation of prioritisation system:	
Yes	5 (23.8%)
No	15 (71.4%)
I do not know	1 (4.8%)

### Interviews & documents analysis

Seventeen pharmacists were interviewed from 16 organisations; two pharmacists were interviewed as a group interview. One interview was subsequently excluded as the organisation used a pharmacy services prioritisation document that prioritised tasks to be undertaken by pharmacy staff. While their document was well-developed, it did not focus on identifying higher risk patients to be reviewed by pharmacy team members. Of the 15 included interviews, ten were from organisations in England and five were from Scotland. Eleven interviewees shared their patient prioritisation systems, screen shots, or prioritisation criteria for document analysis.

Different pharmacy team members participated in the interview from each organisation. In total, there were four mental health lead pharmacists, three chief pharmacists, three mental health clinical pharmacists, two mental health pharmacy managers, one deputy chief pharmacist, one lead pharmacist for digital medicines, one pharmacist in electronic prescribing, one clinical pharmacy team leader for the inpatient mental health services, and one advanced mental health pharmacist specialising in education and training. Some had joint roles such as lead for substance services. Five had predominant roles in developing their system, seven were involved in the development process, and two were not involved in the development of the existing system but in improving it or developing a new system.

#### Types of prioritisation systems

**Fig. 1:**
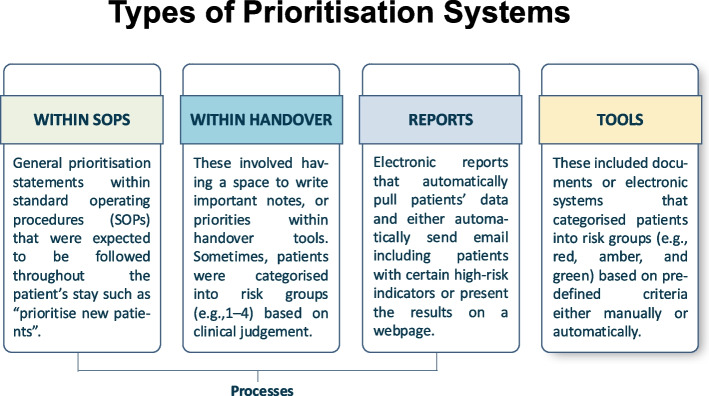
Types of prioritisation systems identified in this study

Patient prioritisation approaches used in the 15 organisations were either guidance within their pharmacy standard operating procedures (SOPs) (*n* = 3), prioritisation within handover tools between pharmacy team members (*n* = 3), or tools/reports designed specifically to guide patient prioritisation (*n* = 9) (Fig. 1). In this study, a broad definition of patient prioritisation systems was adopted to highlight the variation in complexity and diversity in existing prioritisation practices. Examples of mental health related patient prioritisation criteria used in these prioritisation systems are presented in Table 3.

**Table 3:** Examples of mental health related patient prioritisation criteria used in analysed prioritisation systems

Examples of mental health related criteria
• Mental Health Act related issues (e.g. T2/T3 due, SOAD review)
• New patients prescribed medicines for physical health problems
• Patients who have returned from acute hospital following period of leave for physical health intervention
• High Dose Antipsychotic Therapy (above 100% BNF maximum)
• Increase of a regular psychotropic within 7 days of the last increase (unless as part of a dose titration regimen)
• Rapid tranquillisation prescribed
• More than one antidepressant/hypnotic/anxiolytic prescribed
• More than two mood stabilisers prescribed
• Regular prescription of any anxiolytic for more than 4 weeks • New medication for substance misuse prescribed
• Cross tapering psychotropics
• BPSD when not on a dementia ward
• Self-administration or covert administration of medication
• Patients with swallowing difficulties
• Patients receiving ECT
• Patient in seclusion
• High risk medicines: lithium, clozapine, depot antipsychotics, Zuclopentixol Acuphase
• Valproate prescribed for women of childbearing potential

*T2/T3* Certificate of consent to treatment/certificate of second opinion, *SOAD* Second opinion appointed doctor, *BNF* British National formulary, *BPSD* Behavioural and psychological symptoms of dementia, *ECT* Electro-convulsive therapy

##### Prioritisation within organisation standard operating procedures

Three England based organisations developed guidance within their pharmacy SOPs to help pharmacy team members in identifying which patients to approach first (Table 4a). These SOPs included general prioritisation statements that were expected to be followed throughout the patient’s stay such as “prioritise new patients”.

**Table 4:** Types and description of prioritisation systems used by interviewed organisations

Type	Organisation/interview number	Service for which patients are prioritised	Scoring	Description	Used by pharmacy technicians?	Years used/year of implementation
a. Prioritisation within the SOP	Oxford Health NHS Foundation Trust	Medication counselling Discharge planning Enhanced medication reviews SOAD^a^ support	None	Enhanced services described with the SOP for specific patient groups	Yes	4
	Lincolnshire Partnership NHS Foundation Trust	Pharmacy review	None	An SOP including primary, secondary, and tertiary tasks	Yes	1–5
	In.13	Pharmacy review	None	Priority statements within the SOP	Not sure	1–5
b. Prioritisation within handover	NHS Borders	Pharmacy review	Level 1–4	A template in the system that is completed then a score is given based on pharmacist’s judgment	Yes, with scoring criteria (Level 1–3)	3
	Avon and Wiltshire Mental Health Partnership NHS Trust	Pharmacy review	None (traffic light system under development)	A Microsoft Excel handover sheet with a priority column where pharmacy staff can write anything they believe makes a patient priority	Yes	1–5
	In.12	Pharmacy review	None	Four handover tools used in four different sites and a new tool is under development	Yes	-
c. Prioritisation reports	Cornwall Partnership NHS Foundation Trust	Pharmacy review	None	An electronic system pulls and highlights specific patients’ risk indicators in a webpage that is updated every hour	Yes, simpler version	2
	NHS Forth Valley	Lithium & clozapine review	None	Two e-reports, one highlights patients with abnormal lithium or clozapine blood levels (calculate ranges based on age), the other highlights prescribing data for patients on clozapine or lithium	Yes	1–5
	North East London NHS Foundation Trust	Pharmacy review	None	Three automatic reports are sent daily and one twice daily to the pharmacy team each including patients with a specific risk indicator	Yes	2018
	NHS Lanarkshire	Pharmacy review	None	Four automatic reports are sent to the pharmacy team each including patients with a specific indicator	Yes	1–5
d. Prioritisation tools	Derbyshire Healthcare NHS Foundation Trust	Pharmacy review	Traffic light	A word document including red, amber, green list of risk indicators	Yes	1
	In.5	Pharmacy review	Level 1–4	Paper based categories for high-risk patients, used daily by pharmacists & reported electronically. Pharmacy technicians have a separate pharmacy referral tool	Yes, different tool	6–10
	Ayrshire & Arran	Pharmacy review	Traffic light	An automatic scoring system that sends a daily report of patients’ scores	Yes	2016
	South West Yorkshire Partnership NHS Foundation Trust	Pharmacy review	Highlights high risk patients	A list of key priorities to identify patients who need additional pharmacy support	Yes	2003
	Bradford District Care NHS Foundation Trust	Pharmacy review	Traffic light	A word document including red, amber, green list of risk indicators	No	2020

Organisations were named in the table if the representative pharmacy professional consented to waive anonymity on behalf of the organisation. Otherwise, the interview number was used instead

^a^SOAD second opinion appointed doctor

##### Prioritisation within handover tools

Three organisations prioritised patients through pharmacy team handover documents that were used by pharmacists and pharmacy technicians (Table 4b). Handover documents generally relied on clinical judgement when prioritising patients and one organisation assigned scores to patients based on clinical judgement for pharmacists and pre-defined criteria for pharmacy technicians.

##### Prioritisation reports

Four organisations developed electronic reports that highlighted patients with specific ‘high risk’ indicators to guide the pharmacy team in prioritising patients (Table 4c). Three organisations used an electronic system that emails daily reports to pharmacy team members, each including a list of patients with a certain high-risk indicator, one of which was only focused on two medications (lithium & clozapine). One organisation had a webpage that displayed all patients on a ward and highlighted a number of risk indicators. The webpage was designed to show information succinctly with extra details appearing when staff moved the cursor over an item.

##### Prioritisation tools

Five organisations reported using internally developed prioritisation tools that categorised patients into risk groups (Table 4d). All tools had lists of risk-indicators and relied on pharmacy team members manually scoring patients except one that was completely automated.

#### Development of prioritisation systems

Prioritisation systems were mostly developed in-house by senior/lead pharmacists, sometimes with the help of other pharmacists, pharmacy technicians or project managers. Electronic prioritisation systems (*n* = 5/21) involved additional participation of data analysts, informatics pharmacists, or information technology teams in their development.

**Fig. 2:**
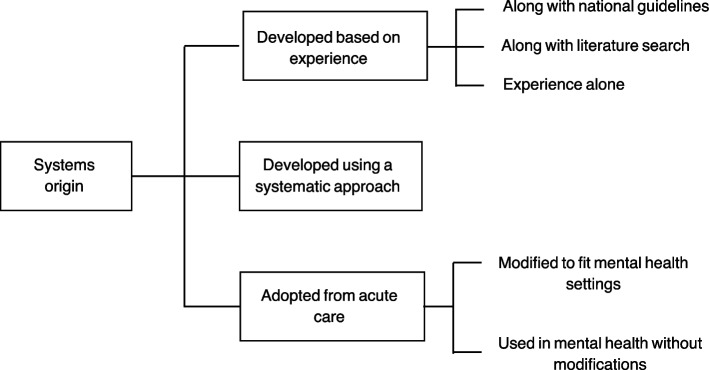
Origin of prioritisation systems

Prioritisation systems were mainly developed internally by staff members who used their experience to design the system and select prioritisation criteria (*n* = 18/21) with a few adapted from acute care organisations (*n* = 2/21), and one developed systematically through a series of stages (*n* = 1/21) (Fig. 2). Although most prioritisation systems were developed based on experience, some also considered national guidelines (*n* = 3/19) or existing prioritisation systems used in acute care organisations (*n* = 1/19). Interviewees discussed a lack of patient prioritisation tools in mental health which could be adopted and limited evidence of risk indicators for medication related problems.

“As for actually evidence for what constitutes a high-risk medicine for pharmacy review, I don’t think that exists.” (In.9).

#### Reasons for developing prioritisation systems

**Fig. 3:**
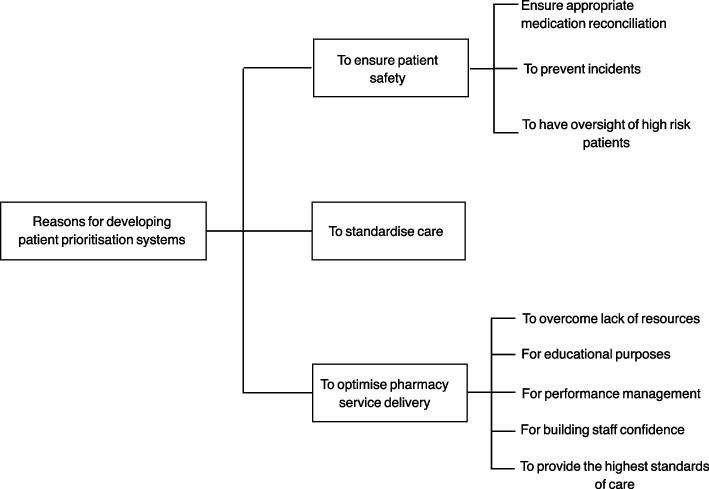
Reasons for developing patient prioritisation systems

Patient prioritisation systems were generally established to standardise care, ensure patient safety, and optimise pharmacy service delivery (Fig. 3). Optimising pharmacy services was the main driver for prioritisation system development. Participants discussed how staff shortages and time management issues had obstructed their ability to see all patients every day and driven them to look for tools to guide them into which patients to see first.

“…with increasing workforce demands and recruitment and retention is increasingly challenging, our time is very pressured. So we need to make sure that we are maximising the efficiency of our clinical services to make sure that the patients get the most safe and efficient care …” (In.2).

One participant explained that the system was needed to direct limited resources during the COVID-19 pandemic. Some participants reported initiating patient prioritisation following the introduction of electronic prescribing at their organisations due to the ease of collecting and reporting information. Prioritisation systems were also seen as educational tools that supported junior or new staff or those new to mental health care.

“We were finding over the last few years, when we have new pharmacists coming into the trust, that they struggle to prioritise the patients that they need to. And often the most unwell patients we weren’t aware of as a team, or we weren’t prioritising them in terms of they needed to be seen a certain number of times a week.” (In.15).

Some participants believed that using prioritisation systems would help pharmacy team members gain more confidence in their work and reduce concerns associated with not being able to see all patients every day. Prioritisation systems were also reported to be developed to help in measuring and benchmarking performance in order to improve and optimise pharmacy service delivery.

“…we can demonstrate to our organisation the value for money of pharmacy service so it’s something we can actually benchmark and demonstrate delivery against.” (In.2).

Participants also discussed unwarranted variation in pharmacy service delivery at both team level and individual level and highlighted the need for standardisation as a reason for system development.

“.. we were consistently getting variations in the pharmacy service based on informal feedback from the service providers but also based on our incident reports. We felt that actually having a standardisation of care would be absolutely essential to make sure that all of our pharmacists were working at the same base level.” (In.2).

Patient safety concerns were another driver of development of patient prioritisation systems. Some participants reasoned that a system was needed to ensure oversight of patients with higher risk of drug related problems. Others discussed the need for prioritisation to ensure new patients received appropriate medication reconciliation:

“One of the things is making sure that new patients, that medicines reconciliation has occurred correctly and if there are any issues arising from that reconciliation that they are dealt with.” (In.13).

Additionally, one participant discussed an incident and the national alert for lithium as key reasons for developing their prioritisation system.

“There had been a patient who had been admitted with lithium toxicity through our out of hours service. It transpired that they had an acute kidney injury and an infection in the kidney as well. But they were also displaying signs of lithium toxicity which weren’t picked up on. The patient came in, the lithium level wasn’t taken. They were started on some renally toxic antibiotics…which obviously then pushed the lithium level up even further causing more damage and the patient came to severe harm from that.” (In.8).

All interviewees believed their prioritisation systems successfully achieved their aims, sometimes with additional benefits. Such benefits included having information records to help pharmacy staff when working on wards they were not familiar with or when a patient was transferred from another ward. Prioritisation systems were found to be valuable for managers in allocating resources effectively and providing evidence to secure funding and support expansion of resources.

#### Implementation of prioritisation systems

Three organisations completed a small pilot testing phase in one or more wards before implementing their prioritisation system. Numerous organisations implemented their prioritisation systems following informal staff user training and some provided guidance on how to use the prioritisation system within their SOPs or during induction for new staff, whereas one organisation opted for a continuous training approach.

**Fig. 4:**
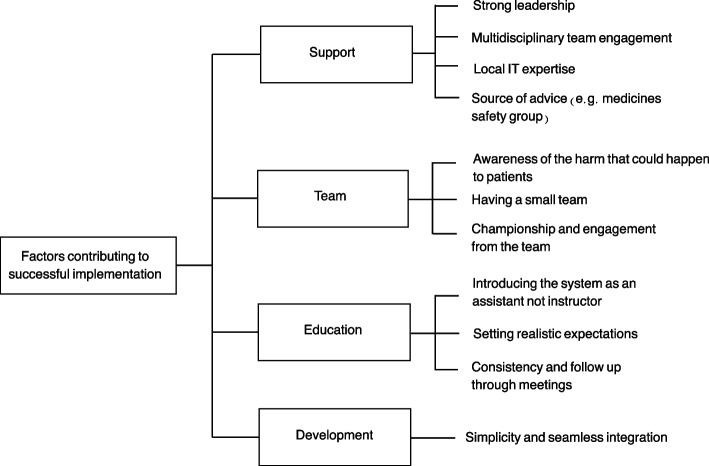
Factors contributing to successful implementation

Several factors were felt to contribute to the successful implementation of prioritisation systems (Fig. 4) including having strong support from a leader and the multidisciplinary team, a medicines safety group, and readily available technical support.

“We have a medicine safety group which is overarching and which feeds into the clinical governance working plan. They are quite high profile. That has also helped to develop change because that is another forum to discuss these things and ask for advice and support and other peoples’ opinions.” (In.8).

Some interviewees discussed the importance of having a team that recognises the potential for medication related harm and good staff engagement with the prioritisation system.

“So I suppose the most important factor is the engagement and understanding of the individual pharmacist who’s following the standards.” (In.13).

Clear messaging regarding the purpose and expectations of prioritisation system use was a factor in successful implementation. One interviewee emphasised the importance of setting realistic expectations, as pharmacy staff may misinterpret the purpose of the system, believing they must see a certain number or category of patients every day, leading to disappointment if they can’t fulfil this expectation. Communicating to the pharmacy team that the purpose of prioritisation was to boost confidence and reduce stress by providing oversight of unwell patients and facilitating time and workload management was therefore crucially important.

“We introduced this tool, and … there was anxiety about leaving your shift before seeing these patients [high and medium risk]. In actual fact, these patients were still … the same patients as last week, but the tool then just reveals that these patients are at that certain risk score.” (In.9).

However, implementation shortcomings within mental health organisations were described by interviewees and included inadequate training, variation of use in practice, lack of understanding, and lack of parity.

“They are very much, sort of, focussed on getting the acute hospital up and going first with it and then we’re [mental health] a side line, unfortunately.” (In.1b).

#### Characteristics of prioritisation systems

All systems depended on pharmacy team members’ expertise along with the guidance provided by the system, except one completely automated system in which pharmacy members were unable to manually change the risk scores it produced. A pharmacy report was generated by this automatic system on which pharmacy members could highlight any issues. For example, they could report on issues that required follow up in a lower-risk patient.

“Within our system, we’re able to highlight within that report any patients that need reviewed…So there’s other ways in which, along with the prioritisation tool, pharmacy staff can highlight for themselves as a reminder..” (In.9).

Clinical judgement was viewed by most participants as an essential element of the prioritisation process that complemented the system when used appropriately.

“We expect pharmacists, and encourage pharmacists to use their own clinical judgement, and you cannot account for all circumstances within any document, if it’s an advisory statement. We expect people to follow it, however I like to think that Senior Pharmacists who disregard it, would have a good reason for doing so.” (In.6).

Although prioritisation systems varied greatly between organisations and some were paper-based, they all captured the outcome following completion of the system electronically in a Microsoft Excel sheet, shared drive, or other means of electronic data storage. This allowed pharmacy team members to have a quick and comprehensive overview of wards and patients prompting timely interventions.

“…we get a daily report that covers all patients on it and it pulls out the current pharmaceutical care plan that is in the notes. So, at a glance, we can see for the team, the wards that they cover, patients who have been prioritised, what their priority code is and it flags if they’re overdue for review.” (In.5).

All patient prioritisation systems were used on patient admission to hospital and then reviewed throughout the patient’s stay, except one system that was reported to be used twice weekly as it focused on two medications only. Although systems were used at every visit to the ward, the frequency of visits depended on the ward type and pharmacy coverage. All systems were used within inpatient units with three extending to other areas such as psychiatric liaison services, home treatment teams, and outpatient settings.

The use of the prioritisation system was considered compulsory or obligatory by most interviewees. Only three affirmed that the use of the prioritisation system was not compulsory and their reasons for this included a concern that it might hinder the use of clinical judgement or they were awaiting a new electronic prioritisation system.

“it’s by all means not… made compulsory. It’s a tool ……… in the same way that many other ways that you would be reviewing a patient. It’s not compulsory to review the patient’s bloods, but it is very often a safe thing to do” (In.9).

None of the prioritisation systems investigated in this study assigned patients to pharmacy members based on expertise or pay band. Many interviewees believed that there was no need for that as long as there was appropriate supervision or peer support/review of pharmacy staff at the organisation.

“…I don’t necessarily think that all high-risk patients should necessarily be seen by a more advanced pharmacist but yes, I suppose I strongly feel that there should be a team and there should be access to that in terms of expertise.” (In.5).

Some believed that such approach would be impractical due to the geographical distance between wards and insufficient staff and some expressed concerns that this approach may impact the learning and development of pharmacy members.

On the other hand, some interviewees had positive opinions about assigning patients to pharmacy members with more expertise depending on factors such as adequate staffing and system sophistication.

“But I guess, with things we report on at the moment, rapid tranquilisation and clozapine, you know, we’d expect even relatively newly-qualified pharmacists, as long as they’d been inducted into mental health, to be able to know what to do in those circumstances. So, I guess, the complexity of what we’re asking it at the moment isn’t high. But if we did more with it in the future, then that’s something we might want to do.” (In.11).

Some organisations discussed upskilling pharmacy technicians with future plans to assign low risk patients to pharmacy technicians and high-risk patients to pharmacists, and having some “prioritisation pharmacy technicians” trained to prioritise patients for pharmacist review.

#### Limitations of prioritisation systems

**Fig. 5:**
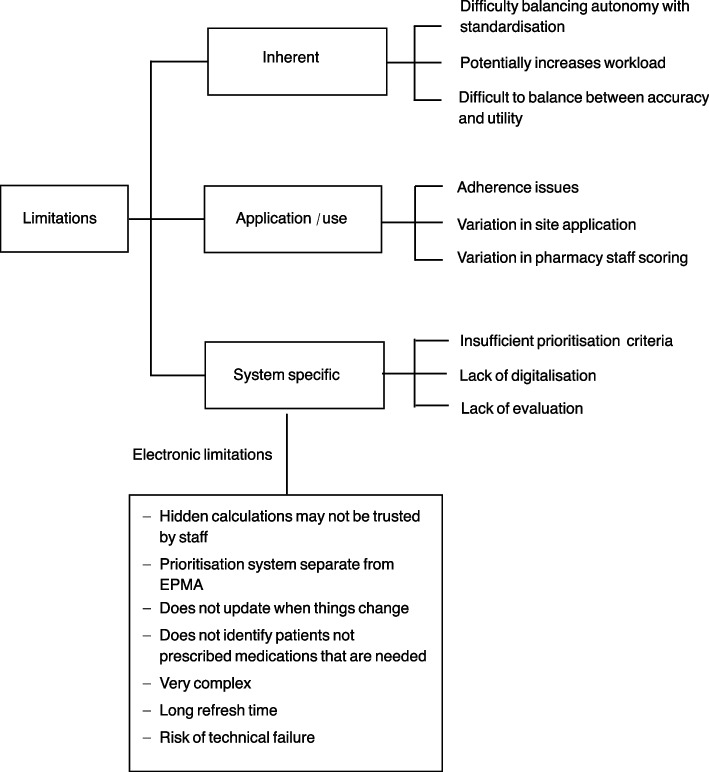
Limitations of prioritisation systems

Interviewees discussed some challenges with the use of prioritisation systems. Some of these challenges were inherent (i.e. they exist naturally and are fundamental to the prioritisation process), some related to the use and application of the system, and some were specific to the system used at each organisation (Fig. 5).

A chief inherent drawback was balancing clinical autonomy and standardisation. Interviewees felt that having standardised prioritisation criteria was important to ensure all patients received the same level of pharmacy service. However, they expressed that it might be difficult to enforce the prioritisation criteria on highly experienced staff whereas more junior pharmacy members may not use their clinical judgement alongside the criteria. For example, one interviewee expressed concerns with junior staff hesitating in going beyond prioritisation tool criteria to categorise patients based on their own clinical judgement.

“it’s very structured so sometimes it doesn’t allow for that nuanced thinking and I think sometimes more junior pharmacists [are] probably a little bit scared to go outside of this because they think that, oh, well, we’ve clearly defined what a ‘red’ patient is, so this patient I’m a little bit worried about I’ll just leave as ‘amber’ or ‘green’. But actually, they might want them to be seen daily because of another reason.” (In.15).

Other disadvantages discussed included lack of a standardised criteria for prioritisation and the concern of the use of prioritisation systems for performance management.

“…there’s always that chance that trusts will… people, particularly like boards, chief executive level, management level, will expect you to baseline against this or audit against this …you’ve got to be careful that it can be overly prescriptive and not allow for that clinical interpretation and you’re also then held accountable for it….” (In.2).

Another drawback discussed was that prioritisation systems could increase workload. Pharmacy members reported that when having a system in place to alert them of ‘high-risk’ patients, they have to see those patients and provide them with the care needed which may reduce time available for other tasks.

“I think obviously the only drawback is once you have seen these patients you can’t un-see them so you have got to do something about them. Whereas if you were just waiting on people phoning you to tell you somebody was in you would be reacting. Whereas this you are proactively finding patients so that obviously potentially increases your workload.” (In.8).

Lastly, the effectiveness of prioritisation systems was deemed limited by the lack of finely balanced prioritisation criteria, which was considered essential for successful implementation. For example, some discussed that prioritisation systems were not holistic and did not include all possible risk indicators whereas others stated that adding more risk indicators might lead to all patients being prioritised.

“I suppose as we keep adding things in, the risk is that everybody is going to be identified as requiring that increased support.” (In.10).

Several disadvantages were related to specific prioritisation systems such as not having pre-set prioritisation criteria, insufficient prioritisation criteria (e.g. polypharmacy not included), having a paper-based system, and lack of validation to ensure the effectiveness of the system. Specific drawbacks were related to electronic systems, for example, electronic prioritisation systems were considered too complex to edit or fix making them unreliable.

When asked about the risk of pharmacy staff choosing particular patients for review (e.g. choosing easy patients), most interviewees believed there was no such risk. One interviewee, however, thought this was unlikely with an automated scoring system but possible when scoring manually.

“It depends, doesn’t it, if it’s an automated system, then the pharmacist is forced to see patients, and so they wouldn’t necessarily be able to pick and choose… if it’s manual, then it requires two people, I think it requires one to triage and then another to follow that triage system. If it’s based to one person filling out the tool manually and then following it, then I think there is risk of that, isn’t there?” (In.12).

Interviewees were asked whether they believed prioritisation of patients might risk oversight of some patients who would benefit from pharmacy input. Overall, interviewees felt that the benefits of prioritisation systems outweighed the risks. Some interviewees stated that the risk of oversight was present independently of the system, and the system actually reduced this risk. One interviewee believed that this risk was mediated by the experience of the individual who assigned prioritisation to a patient.

“There is that risk, isn’t there, but it’s weighing up whether seeing 95 per cent of the more acutely unwell patients outweighs missing those five per cent, you know, and it relies on the experience of those that are triaging those patients, because it needs somebody to triage in the first instance, so … you need that experience to be able to do that properly.” (In.12).

Some believed there was a risk with their current prioritisation system as it lacked some high-risk indicators such as physical health issues and or it did not identify patients who had not been prescribed medications that they required. On the other hand, others believed that there was no risk as long as the prioritisation system was used appropriately alongside other things, or as long as all patients are seen weekly.

#### Evaluation of prioritisation systems

**Fig. 6:**
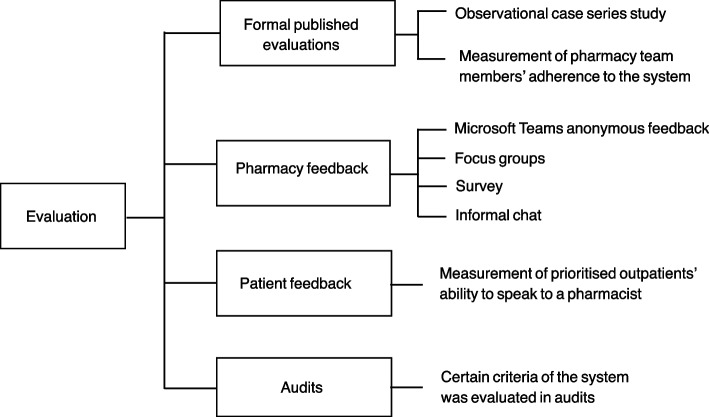
Evaluation of prioritisation systems

The majority of interviewees (*n* = 11/15) described some type of evaluation of their prioritisation system in varying levels of complexity (Fig. 6). Additionally, one interviewee explained that their prioritisation system was validated in acute care prior to adaptation. A few organisations had not completed any evaluation whereas some expressed their interest in future validations.

Two organisations conducted and published formal evaluations. One was based in Scotland and measured adherence to the ranking system using a comparison study. Pharmacy students scored patients independently and compared their ranking with pharmacists’ assessment then conducted focus groups to understand the factors contributing to non-adherence with the prioritisation criteria. The other organisation conducted an observational case series study which resulted in changes to their prioritisation system’s criteria.

Feedback from prioritisation systems users was on the whole, positive and interviewees described how systems enhanced pharmacy services and improved patient outcomes. Moreover, one interviewee commented that they received fewer patient safety medication incident reports after implementing their prioritisation system.

Barriers to evaluation included insufficient time, having a simple system (e.g. does not record pharmacy actions), lack of key performance indicators in pharmacy to use as measures, lack of pre-intervention data, and difficulty in measuring patient outcomes such as rates of drug related problems.

“data collection is always tricky, because what you measure, and a lot of pharmacy systems essentially measure activity and not outcomes. And measuring outcomes in a real world environment is also very hard, because there’s so many factors that affect it.” (In.6).

## Discussion

This multi-method study has explored for the first time patient prioritisation approaches used by UK mental health pharmacy teams. Findings revealed that a significant proportion of mental health organisations that responded to the questionnaire reported implementing patient prioritisation systems or expressed interest in future adoption of such systems. These efforts in optimising pharmacy services and medicines management align with NHS recommendations to improve the quality of care in mental health [19]. Interviewees found prioritisation systems valuable in ensuring patient safety, standardising care, and optimising pharmacy services. However, heterogeneity was observed between prioritisation systems in terms of their development, configuration, and application, often accompanied by local, limited scope evaluations.

Prioritisation systems varied greatly from simple guidance to complex electronic systems, were mostly developed based on expertise, and none were validated based on patient outcomes which is comparable to findings in acute care [24]. Prioritisation systems identified in this study were either simple guidance within the pharmacy standard operating procedures, handover tools between pharmacy team members, or tools/reports designed explicitly to prioritise patients. Additionally, some systems were electronic and automatically highlighted patients with certain high-risk indicators or calculated risk scores. While standard operating procedures and handover tools are invaluable, incorporating patient prioritisation within them may not be ideal as these are generally site specific. Prioritisation reports (electronic reports that highlighted patients with specific ‘high risk’ indicators) could be more transferrable, but they heavily rely on clinical judgement as they do not score or categorise patients, and these can only be used in hospitals employing electronic health records. Prioritisation tools, however, may provide more flexibility in design and application. They can be electronic, paper-based, or both and could be designed for use in multiple sites which is particularly important for mental health units considering their geographic diversity. Future research could focus on developing a patient prioritisation tool that could be widely implemented across different mental health sites in the UK. Additionally, organisations could measure patient outcomes prior to implementing a prioritisation tool to enable a before-and-after evaluation similar to previous research in acute care [36].

Prioritisation systems identified in mental health organisations included several mental health related prioritisation criteria highlighting the different challenges in mental health wards compared with acute care. For example, issues related to mental health law, dosing and combination therapy of psychotropics, rapid tranquillisation, electroconvulsive therapy, and substance abuse none of which was included in the adult complexity tool for pharmaceutical care (ACTPC) developed for acute care [37]. Comprehensive systematic research should identify appropriate patient prioritisation criteria for use in mental health wards. Insights for developing and implementing prioritisation systems were derived from the interviews and organised in a table of key features to consider for a prioritisation system **(**Table 5**).** Additionally, factors influencing the successful implementation were also summarised in the results, many of which align with the Medical Research Counsel guidance for developing and evaluating complex interventions [38] such as ensuring consistency and follow up. These features and factors could be used by mental health organisations to make informed decisions when developing and implementing patient prioritisation systems. Ideally, any planned prioritisation system should undergo a phased evaluation using both quantitative and qualitative approaches [39]. The use of mixed-methods aid researchers in exploring contradictory results and ensure a holistic understanding of the intervention outcomes [38]. The evaluation process could follow the Medical Research Counsel framework for evaluating a complex medical intervention as this frequently used framework was rigorously developed to help researchers evaluate interventions using appropriate methods [40].

Despite many UK mental health organisations reporting the use of patient prioritisation systems, the adoption rate appears lower than acute care where around half of organisations that responded to the questionnaire indicated using a system in 2020 [24]. This variation may not be unexpected given an observed lack of parity between mental health and acute care services especially in funding and resource allocation [41] which was reaffirmed by interviewees in our study. Although limited workforce capacity was considered to be a barrier to implementing prioritisation systems by questionnaire respondents, it was also considered to be a driver for developing such systems by interviewees implying that executing prioritisation systems may be associated with short-term workload challenges but could bring long-term benefits. A recent report by the UK National Audit Office reported that staff shortages continue to be major obstacle preventing advancement of services, and plans to achieve a full parity of esteem for mental health services will at least be delayed one year due to COVID-19 pandemic [3]. This emphasises that organisations may not be able to rely on future staffing improvements and could instead start implementing innovative approaches to enhance their services [2].

Our findings indicated that pharmacy teams across many organisations prioritise their services or which patients receive them, if not using a formal prioritisation system, then by simpler means such as prioritising certain wards (e.g. with high patient turnover) or newly admitted patients. The use of formal patient prioritisation approaches may be more advantageous as it ensures standardisation as well as the use of a rigorously developed prioritisation criteria, the impact of such approaches was reported in a systematic review of pharmaceutical patient prioritisation tools [25]. Prioritisation criteria should ideally be developed based on both evidence and expertise, yet most organisations relied on expertise while developing their systems. Lack of evidence on risk factors for medication related problems in mental health might be a reason for over-reliance on expertise- a claim supported by our findings and a recent systematic review of the literature [42]. The review summarised existing evidence on risk factors for drug related problems in mental health units identifying several risk indicators such as increased number of prescribed medications but has also underscored the clear necessity for more research using robust methodologies [42]. In addition, questionnaire respondents and interviewees alike discussed the lack of a patient prioritisation system developed for mental health pharmacists to adopt. While two organisations participating in this study used a prioritisation system developed for acute care, other interviewees believed such tools will be difficult to use in mental health settings due to different priorities and patterns of medication related problems. Two other organisations modified acute care systems to ‘fit’ mental health prior to implementing them. Several considerations for developing and implementing prioritisation systems in mental health were identified in this study underscoring the need for mental health specific prioritisation systems. First, prioritisation criteria identified in existing prioritisation systems differ from what has been reported in acute care [24] with several mental health specific criteria such as those related to the Mental Health Act and antipsychotics. Second, mental health wards can be located in remote or distant locations limiting staff members' ability to move across sites. Third, unlike acute care, some wards may have medically stable patients requiring fewer pharmacy visits. Whilst evidence on high-risk indicators in mental health remains limited, consensus methods could be a salient strategy to develop patient prioritisation systems. Consensus methods are commonly used approaches when empirical data is limited as they facilitate the construction of evidence by gathering agreement among a panel of experts in the field [43, 44].

One concern raised about patient prioritisation approaches was the challenge in achieving accuracy when categorising patients. The aim of prioritisation approaches, however, is not to achieve accuracy but rather to categorise patients based on factors such as their needs, expected benefits, severity, and urgency [45]. This supports the pharmacy team to manage their workload by making decisions based on a standardised pre-defined criteria [25]. The positive impact of pharmaceutical patient prioritisation systems was observed by interviewees in this study and previously documented in acute care [24, 25, 46]. Another concern raised was the difficulty in balancing between autonomy and standardisation as some interviewees showed concerns about systems being too restrictive preventing clinical judgement whereas others over-relied on clinical judgement and were concerned about variation in use of their system. Similar concerns were reported in acute care where variation was described between pharmacists’ scoring depending on [24] or regardless of [47] their level of experience. Additionally, similar findings were described with a medication optimisation system implemented in primary care [48, 49]. The importance of having a room for clinical judgement was highlighted in a study that measured pharmacist’ attitudes towards a patient prioritisation tool in an acute care hospital where pharmacists explained that they sometimes score patients based on staffing levels [47]. For example, scoring patients higher than their actual score to ensure they are seen by a pharmacist when there is no pharmacy technician to see them [47]. In acute care, however, several organisations assigned pharmacists to patients with certain risk levels based on their band or experience [24]. This phenomenon was not seen in our study where pharmacists considered it impractical given the workforce shortages and the distances between organisation sites. Additionally, similar to acute care, some interviewees felt it could negatively affect the learning and development of less experienced staff [24]. Last, prioritisation systems were thought to increase workload through directing pharmacy staff to proactively identify and resolve drug related problems. However, this highlights the burden of staff shortages, which was discussed by many interviewees, as without a system in place to notify pharmacists, critical issues may go unnoticed leading to patient harm. This underscores a pressing need for additional staffing for mental health organisations along with a strong emphasis on the retention of pharmacy staff. Concurrently, researchers should focus on identifying innovative strategies aimed at effective resource allocation and optimising service delivery.

Key strengths of this study included applying different methodologies that facilitated a comprehensive investigation at a national scale. The questionnaire provided exploratory insights enabling us to quantify the use of patient prioritisation systems and explore reasons behind non-adoption. The interviews allowed us to explore prioritisation approaches in depth for the first time in mental health care. The document analysis allowed visual inspection of the prioritisation systems and complemented the interviews enabling direct comparisons between verbal descriptions and the actual content of systems. Another strength was that the questionnaire and interview guide were both rigorously developed and piloted to increase their validity. Last, the high response rate achieved in this study ensured good representation of national mental health organisations. Nonetheless, this study has some limitations. Despite efforts to clearly define patient prioritisation systems in the questionnaire to avoid confusion [24], some respondents struggled to grasp the concept of a patient prioritisation system. Yet, analysis of open-ended questionnaire questions was performed to enhance the accuracy of the results and respondents were contacted for further clarifications when deemed necessary. Semi-structured interviews are susceptible to a number of biases including researcher bias, organisational attribution bias, and confirmatory bias. To mitigate these, the interview guide was carefully developed, piloted, and the first few interviews were analysed to ensure avoidance of leading questions. Furthermore, the interviewer (FQA) was not a mental health pharmacist and had no prior experience in the UK which ensured a fresh perspective during the interviews and minimised researcher’s bias. Last, data from the questionnaires and document analysis complemented the interview findings ensuring greater depth and credibility of the analysis. An additional point to be considered is that an interview with a single representative from an organisation may not fully represent the perspectives and experiences of others as observed from the group interview in this study.

## Conclusion

This study identified 21 UK based mental health organisations using systems to prioritise patients for clinical pharmacy services. Several patient prioritisation criteria were specific to mental health care including issues around psychotropic drug use and mental health law, use of rapid tranquillisation and seclusion. Similar to findings in acute care, patient prioritisation systems were reported to be valuable in ensuring patient safety, standardising care, and optimising pharmacy services [24]. However, there was significant heterogeneity in systems used and the majority were simplistic in design (e.g. had no or minimal prioritisation criteria), developed solely based on existing expertise, and lacked formal evaluation warranting future research to focus on developing an evidence-based pharmaceutical patient prioritisation system. Several points must be considered when developing a mental health specific system such as the geographic dispersion of staff, variations in staffing across different inpatient sites, and the importance of identifying mental health specific patient prioritisation criteria.

**Table 5:** Features of a good prioritisation system

Feature	Example Quote
Electronic	“I think the biggest addition will be moving electronically.” (In.2)
Has ranking system	“…because at the minute it’s just a list, and if anyone triggers any of them then we will do it. But I think something that would give us some sort of scoring or ranking for them [will make the system better]” (In.10)
Categorise patients using colours	“I think kind of the colour coding system will probably be quite good” (In.7)
Incorporate primary care data	“I think the future will be … potentially looking at primary care data, and that would indicate maybe any missed medicines that aren’t currently prescribed.” (In.9)
Integrated with the electronic prescribing system	“It is external to the system, it would be great if it was somehow included in the systems that it draws data from, so that it could feed back into those systems. It would be great if you could do things from that as well, so you could like I say tick things off, but also maybe action things on EPMA based on that.” (In.4)
Simple yet inclusive	“Simpler is always better in some respects, but unfortunately most things aren’t quite that simple. We have three areas of tasks, with probably six or seven tasks in each area, which is then 18 tasks that people have to think about. So less would be better, but if you lose detail, you tend to get confusion and people interpreting the said tool differently.” (In.6)
Include a worked example	“I sometimes wonder whether it would be nice to have like an example with a worked method of this is what this means, this is what you might do about it, so that might be useful as well.” (In.4)
Interactive	“What we wanted to do was also give that element of a pharmacist or an MMT [Medicines management technicians] being able to make a clinical call to make something … [categorise a patient based on clinical judgement] that isn’t on the list.” (In.2)
User friendly	“I think if people had to do more writing, they would not have done [used the system].” (In.2)
Has glossary for abbreviations	“I think some way to explain the various columns might be nice because for space reasons quite a lot of it is very heavily abbreviated so that it fits in, so a key or glossary or some way to explain those things would be useful.” (In.4)
Piloted in different wards	“It would have been nice to try it out implementing-wise on a more acute ward where the more turnover on the med recs was present. We didn’t get to trial it out maybe in all the different settings I would have liked to before we launched so I think that would have been really useful to have a better trial run.” (In.2)
Introduced with proper education	“So on reflection on how we would do differently … would have been an opportunity to give a clearer indication as to kind of how it should be used.” (In.9) “So I suppose the most important factor is the engagement and understanding of the individual pharmacist who’s following the standards.” (In.13)
Standardised between sites	“The problem with that is that if a patient moves from one area to another, then all of that information doesn’t slot in nicely to the receiving area.” (In.12)
Involvement of the team	“I would rather it be less led by me, and the junior pharmacists kind of take the lead on it, because I feel like there’d be more buy-in from people that way.” (In.15) “Buy in is always important, I think any model that I’ve ever read, and in my own personal experience, I would agree that buy in from the team is the most important. And being involved in the creation of a prioritisation tool while they were here, hopefully means they had a greater buy in to the system” (In.6)

## Supplementary Information

Additional file 1. Microsoft Word (.docx). Questionnaire. This is the questionnaire questions developed and used for this study.

Additional file 2. Microsoft Word (.docx). Interview guide. This is the interview guide developed and used for this study.

## Data Availability

The datasets used and/or analysed during the current study are available from the corresponding author on reasonable request.

## Abbreviations

UK: United Kingdom
MHA: Mental Health Act
NHS: National Health Services
PCNs: Primary Care Networks
BPSD: Behavioural and Psychological Symptoms of Dementia
SOP: Standard Operating Procedures
COVID-19: Coronavirus Disease 2019
SOAP: Second Opinion Appointed Doctor
