# Supplementary material for: Exploring current approaches towards patient prioritisation for clinical pharmacy services in UK mental health inpatient care
Source: BMC Psychiatry. 2025 Jul 1;25:617. doi: 10.1186/s12888-025-06956-4 (PMC12210832; doi:10.1186/s12888-025-06956-4)
Supplement: Supplementary file 1 — Additional file 1. Microsoft Word (.docx). Questionnaire. This is the questionnaire questions developed and used for this study. [file 12888_2025_6956_MOESM1_ESM.docx]

**Additional file.1**

**Questionnaire**

I have read and understood the Participant Information Sheet (version 1, dated 23-03-2022), and I agree to take part in this survey knowing that participation is voluntary.

- - I consent
  - I do not consent

1. Which country is your mental health organisation based?
   - England
   - Wales
   - Scotland
   - Northern Ireland
2. Do you use any system* (process or tool) to prioritise inpatients for pharmaceutical care or clinical pharmacy services within your organisation? This could be in all or parts of your organisation, such as a specific ward or specialty. 
   * A process could be a simple rule or a more complex process that includes a series of steps that guide pharmacy team members in approaching hospitalised patients. A tool could be electronic or paper-based and might include a list of high-risk indicators.
   - Yes
   - No
   - We have used a system before, but currently do not
   - Other ……….…
3. Regardless of your choice above, if you would like to add details, please add them below: (Optional)

…………………………………………………………………………………………..

**If No:**

3. Is your organisation considering/planning to implement a pharmaceutical care or clinical pharmacy service prioritisation system (process or tool) in the future?

- - Yes
  - No

4. Please briefly explain why your organisation is considering/not considering implementing such a system? (Including any barriers or facilitators to implementing such a system)

**If used before:**

3. Is your organisation considering/planning to implement a pharmaceutical care or clinical pharmacy service prioritisation system (process or tool) again in the future?

- - Yes
  - No

4. Please briefly explain why your organisation decided to use this system, and why is it no longer used? (Including any barriers or facilitators to implementing such a system)

**If Yes or Other:**

3. What is the type of the prioritisation system (tool/process) your organisation uses?

- - Electronic system
  - Paper-based system
  - Both electronic and paper-based system

4. What type of inpatient prescribing system do you have in your organisation?

- Electronic prescribing
- Paper-based prescribing
- Both electronic and paper-based prescribing

5. When was the current prioritisation system (tool/process) introduced in your organisation?

- System currently under development
- < 1 year
- 1 - 5 years
- 6 - 10 years
- > 10 years
- I do not know

5. Has your organisation’s prioritisation system (tool/process) been evaluated in any way? (This may include user feedback, impact of key performance indicators, or any other formal evaluation)

- - Yes
  - No
  - I do not know
  - Comments ……

6. We would like to carry out an online/telephone interview to learn more about the prioritisation system (process or tool) used at your organisation, would you be happy for us to contact you regarding that?

- - Yes
  - No

**All:**

7. If you have any comments or feedback about the survey please add them here: …………………………………………………………………………………………..

8. Name:

9. Organisation:

10. Position:

11. Contact details (email, telephone):
