# Supplementary material for: Exploring current approaches towards patient prioritisation for clinical pharmacy services in UK mental health inpatient care
Source: BMC Psychiatry. 2025 Jul 1;25:617. doi: 10.1186/s12888-025-06956-4 (PMC12210832; doi:10.1186/s12888-025-06956-4)
Supplement: Supplementary file 2 — Additional file 2. Microsoft Word (.docx). Interview guide. This is the interview guide developed and used for this study. [file 12888_2025_6956_MOESM2_ESM.docx]

**Additional file.2**

**Interview Guide**

| Date: | Type of interview: (e.g. telephone) | Interviewer: |
| --- | --- | --- |
| Interviewee: | Organisation: | Position: |
| Reference No: | Start time: | End time: |

**Introduction:**

1. Introduce yourself.

2. Introduce the aim of the study:

**Aim:** To explore current approaches and pharmacy staff experiences of patient prioritisation for inpatient mental health clinical pharmacy services.

3. Introduce the objectives of the interview:

- - - - To identify characteristics of the prioritisation system (tool/process) and its development process.
      - To ascertain the impact of the system and any evaluation or validation.
      - To explore implementation and pharmacy staff experiences using this system.

5. Ask interviewee whether they would like to ask anything before the start of the interview.

6. Signal the start of the interview and start recording.

**Start recording**

**Background questions:**

1. Could you please tell me about your role, what does it involve and how are you connected to the prioritisation system (tool/process) we are discussing?
2. Can you tell me about the structure of your inpatient services? e.g. number of beds, and pharmacy team provision.
3. How long have you been working in mental health?

**Tool background:**

1. Could you tell me why a system (process/tool) was/is needed to prioritise patients at your organisation?
2. Could you describe its development? **Prompt:** When, and who developed it? What is the tool based on? (evidence, data). What were the steps involved in developing it?

**Tool details:**

1. What do you know about the system implementation? **Prompt:** How long did it take and who was involved? Was there any training for staff prior to the use of the system?
2. How was implementation achieved and what factors influenced how successful this was? What would you do differently next time?
3. Can you describe the system and tell me about its contents? **Prompt:** Name of the system if electronic
4. What does the system (tool/process) aim to achieve?
5. How and where is the system used? **Prompt:** Who uses the system and how does it direct action? Is it compulsory?
6. How often is the system used? and at what stages is it used? **Prompt:** Upon admission, or after medication reconciliation
7. Could you give me an example of how the system is used?
8. Does the system take pharmacy team members expertise into account? e.g. when deciding the final risk scores
9. Does the system assign pharmacy team members to patients based on experience or pay band? or is this done informally or planned for the future?

- if so, are any measures in place to ensure pharmacists with lower experience are given the opportunity to work with different levels of patients in order to gain experience?

1. Have there been any evaluations of the system (tool/process) in your organisations? If so, can you describe them and what the outcomes were? P**rompt:** patient feedback, user feedback, impact of key performance indicators such as medication safety
2. Has the system undergone any modifications after its implementation?

- if yes, what was modified and based on what, did it work/what was the impact?

**Advantages and disadvantages of the tool**

1. Could you tell me about your experience with the system? Do you think it is successful in what it aims to achieve?
2. How useful do you think the system is? Is it effective in improving patient outcomes and optimising pharmacy resources?
3. What additions do you think will make the system better?
4. What do you think will make the system easier to use?
5. Are there drawbacks to the system? e.g. pharmacists choosing particular patients or concerns of missing patients?

**Follow up questions**

1. Is there anything else that you think we should know about the system?
2. Would you recommend other organisations to use a system? What would you tell them?
3. If not provided earlier, would you mind sharing copies of policy documents and any earlier drafts or related documents available? This will add great value to this study. (Address is provided here)
4. Would you like the organisation to be recognised or remain anonymous?
5. Would you mind me contacting you if further clarification on your interview data is required in the future?
6. Are you interested in being contacted for future opportunities involving this project?
7. Finally, would you like to add anything before we finish?

**Thank you very much for your time, this was very insightful, I will stop recording now.**

**Explain what happens next:** The interviews will inform the development of an evidence based prioritisation tool for use by mental health clinical pharmacists.
